# One-Dimensional Nanoscale Si/Co Based on Layered Double Hydroxides towards Electrochemical Supercapacitor Electrodes

**DOI:** 10.3390/nano12091404

**Published:** 2022-04-20

**Authors:** Osama Saber, Sajid Ali Ansari, Aya Osama, Mostafa Osama

**Affiliations:** 1 Department of Physics, College of Science, King Faisal University, P.O. Box 400, Al-Ahsa 31982, Saudi Arabia; 217044956@student.kfu.edu.sa (A.O.); 214110595@student.kfu.edu.sa (M.O.); 2 Egyptian Petroleum Research Institute, Nasr City, P.O. Box 11727, Cairo 11765, Egypt

**Keywords:** Si/Co LDHs, 1D nanostructures, nanofibers, cyclic voltammetry, charge–discharge method, supercapacitors

## Abstract

It is well known that layered double hydroxides (LDHs) are two-dimensional (2D) layered compounds. However, we modified these 2D layered compounds to become one-dimensional (1D) nanostructures destined for high-performance supercapacitors applications. In this direction, silicon was inserted inside the nanolayers of Co-LDHs producing nanofibers of Si/Co LDHs through the intercalation of cyanate anions as pillars for building nanolayered structures. Additionally, nanoparticles were observed by controlling the preparation conditions and the silicon percentage. Scanning electron microscopy, X-ray diffraction, Fourier transform infrared spectroscopy and thermal analyses have been used to characterize the nanolayered structures of Si/Co LDHs. The electrochemical characterization was performed by cyclic voltammetry and galvanic charge–discharge technique in 2M KOH electrolyte solution using three-electrode cell system. The calculated specific capacitance results indicated that the change of morphology from nanoparticles or plates to nanofibers had a positive effect for improving the performance of specific capacitance of Si/Co LDHs. The specific capacitance enhanced to be 621.5 F g^−1^ in the case of the nanofiber of Si/Co LDHs. Similarly, the excellent cyclic stability (84.5%) was observed for the nanofiber. These results were explained through the attribute of the nanofibrous morphology and synergistic effects between the electric double layer capacitive character of the silicon and the pseudo capacitance nature of the cobalt. The high capacitance of ternary Si/Co/cyanate LDHs nanocomposites was suggested to be used as active electrode materials for high-performance supercapacitors applications.

## 1. Introduction

The current global energy situation has become more critical because of environmental problems and climate change. Therefore, the urgent and continued need for clean energy has pushed the development of novel alternative sources of energy that are capable of transforming energy as well as storing energy for longer durations [1,2,3,4,5]. Therefore, there is an urgent need for an abundance of energy storage devices such as fuel cells, batteries, and super capacitors (electrochemical capacitors), which are low cost, environmentally friendly, more effective, and easier to manufacture. The interests in supercapacitors are being stimulated due to the potential connection between traditional capacitors and batteries [6,7,8]. Supercapacitors (SCs), also called ultra-capacitors, are considered to be a new type of energy storage/conversion device due to their high-power density, fast charging/discharging rate, long lifecycle, wide operating temperature range, as well as its maintenance-free and ecofriendly features. Due to its excellent properties, it is used in different application systems such as hybrid electric vehicles, electronic devices, mortar starter, memory backup system, industrial power and energy management [9,10,11].

Generally, supercapacitors or electrochemical supercapacitors can be divided into two major categories based on its charging mechanism [12]. The first one is electric double layer capacitors (EDLCs), which store capacity or electrical energy through the adsorption/desorption between the electrode–electrolyte interface and carbon-based materials. These are used as active materials for fabricating electrodes. The second is pseudo capacitors, wherein energy is based on the faradic process at the surface of the electrode in the electrolyte solution and the charge accumulates through the redox reaction. Conducting polymers and transition metal oxide and hydroxide have been used in pseudo capacitors. Pseudo capacitive electrode materials, especially binary metal oxides or hydroxides, have been proven to be an outstanding specific capacitance material. Recently, transition metal silicate oxide or hydroxides have focused on positive electrode materials due to their higher theoretical capacity, easy synthesis, and low cost [13,14,15]. For example, Zhang et al. synthesized three-dimensional Zn_4_Si_2_O_7_(OH)_2_·H_2_O which were used in supercapacitive device applications [16]. 

Among the binary metal hydroxides materials, layered double hydroxides (LDHs) are promising capacitive materials for many electrochemical processes because they have layered structures that contain positive and negative charges which can act as active sites for electrochemical processes [17]. The positive charges of the nanolayers of LDHs are produced from the presence of di- and trivalent metals in order arrangements inside nanolayers. To neutralize the positive charges of the nanolayers, selective anionic species are intercalated to act as pillars for building the nanolayered structures of LDHs [18]. These structures showed a high performance in different fields such as catalysis [19], magnetic and optical applications [20], lithium-ion batteries [21] and particularly, supercapacitors [22,23]. For supercapacitors [24,25], the stable-layered structures of LDHs can attain the demands of efficient supercapacitors such as long-life cycling at a high current density. For example, a high energy density with 95% retention after 10,000 cycles was reported for CoS/NiCo LDHs [26]. Additionally, a maximum energy density 35 Wh kg^−1^ was observed for NiAl LDHs/Ni-MOF [27].

In recent years, [28], Si-based materials have attracted attention in electrochemical processes because of their high theoretical capacity 4200 mA h g^−1^. Si–C–Cu composites [29] and Si–C microspheres [30,31] which showed an enhanced electrochemical performance. Therefore, many researchers have tried to combine silicon with LDHs for increasing the electrochemical performance, but it was difficult because the structure of LDHs depends on di- and trivalent elements. Recently, Li et al. [32] reported that Si supported on NiAl LDHs increased the performance of anode material in lithium batteries. Additionally, the pseudo-capacitive performance of Ni_3_Si_2_ nanowires was observed by Goh’s group, indicating 54.8 C g^−1^ at 0.5 A g^−1^ in the case it was grown on a Ni-coated Si substrate and 313 C g^−1^ at 0.5 A g^−1^ for Ni foil [33]. In addition, higher specific capacitance (760 F g^−1^ at 0.5 A g^−1^) was observed for Ni_3_Si_2_ nanowires grown on Ni foam by Jiang et al. [34]. A good supercapacitor performance was observed through preparing the single-crystalline Co_2_Si nanowires producing ~983 μF cm^−2^ at 2 μA cm^−2^ [35].

To the best of our knowledge, there is no one working on the applications of Si/Co LDHs for supercapacitors. Therefore, in the current study, series of Si/Co LDHs were prepared and transformed to become nanofibers through the confinement of cyanate anions inside nanolayered structures. The optimized Si/Co LDHs electrode material exhibited excellent electrochemical performance in 2M KOH electrolytic solution; the specific capacitance of Si/Co LDHs electrodes was up to 621.5 F g^−1^ at 2 A g^−1^ and the capacitance retention was approximately 86.5% after 3000 continuous charge/discharge cycles. This study can provide a reference for future studies of morphological control and provide the strategies to enhance the performance of supercapacitor electrode materials. Additionally, nanofibers may be useful for designing and fabricating nano-devices. 

## 2. Materials and Methods

### 2.1. Preparation of Nanostructures

The hydrolysis of urea depends on temperature [36]. By controlling the temperature of the aqueous solution of urea, it decomposes at a slow rate whilst producing ammonium carbonate or cyanate through two stages. During the slow decomposition of urea, the reaction medium is converted from acidic into alkaline, which leads to precipitating the nanostructures of Si/Co LDHs. Therefore, the urea was used in this reaction as a pH controller and precipitant. Series of Si/Co LDHs were prepared after mixing aqueous solutions of cobalt nitrate (0.03 M), urea (0.5 M) and silicon chloride (0.0096 M) under vigorous stirring. The reaction is sensitive for the temperature. Therefore, it was precisely adjusted at 80 °C. Depending on the time of the reaction, series of Si/Co samples were prepared. After 16 h, the sample was separated and washed by the distilled water. This sample was coded by SiCo-1-16. By continuing the heating process, the second sample was separated after 20 h and coded by SiCo-1-20. By further heating for 26 h and 36 h, the third and fourth samples were separated and coded by SiCo-1-26 and SiCo-1-36.

By increasing the molar ratio of Si/Co from 0.25 to 0.33, the fifth sample was precipitated after heating for 36 h of the same procedure. After washing and filtration, the product was dehydrated under vacuum at room temperature. The sample was labeled here after as Si/Co-2-36.

### 2.2. Physical Characterization

The morphology of the prepared samples was determined by scanning electron microscopy (SEM). The JEOL JSM-6330F (Tokyo, Japan) was used for imaging solid prepared materials. By using powder X-ray diffraction a Rigaku RINT 2200 (Tokyo, Japan), the structure of the prepared materials was determined through a source of radiation Cu Kα (filtered) at λ = 0.154 nm with angles between 1.8° and 50°. A Seiko SSC 5200 apparatus (Tokyo, Japan) was used for measuring the thermal analyses of the prepared materials using three techniques (differential thermal gravimetric—DTG; thermal gravimetric—TGA; and differential thermal analysis—DTA). The thermal analyses were measured under a flow of nitrogen and were carried out up to 800 °C with a heating rate of 10 °C/min. In order to determine the functional groups of the prepared materials, Horiba FT-720 (Tokyo, Japan) has used for performing Fourier transform infrared (FT-IR) spectroscopy using potassium bromide discs in the range of 400–4000 cm^−1^. 

### 2.3. Electrochemical Measurements

Electrochemical performance of all the electrodes was examined in a three-electrode system with a Pt sheet, a Ag/AgCl electrode used as a reference and counter electrode, respectively. To fabricate the working electrode firstly, slurry active material was prepared by adding 80 wt% of the Si/Co LDH, 10 wt% of activated carbon (AC), 10 wt% of polyvinylidene fluoride (PVDF) in anhydrous 1-Methyl-2-pyrrolidinone (NMP) and mixed properly using a magnetic stirrer at ambient temperature and the slurry of active material coated on chemically cleaned nickel foam of 1 × 1 area with mass loading at approximately 1 mg before drying in an oven at 90 °C for 12 h. All electrochemical measurements were carried out in 2M KOH aqueous solution using CV and CD analysis.

## 3. Results and Discussion

### 3.1. Scanning Electron Microscopy

**Figure 1:**
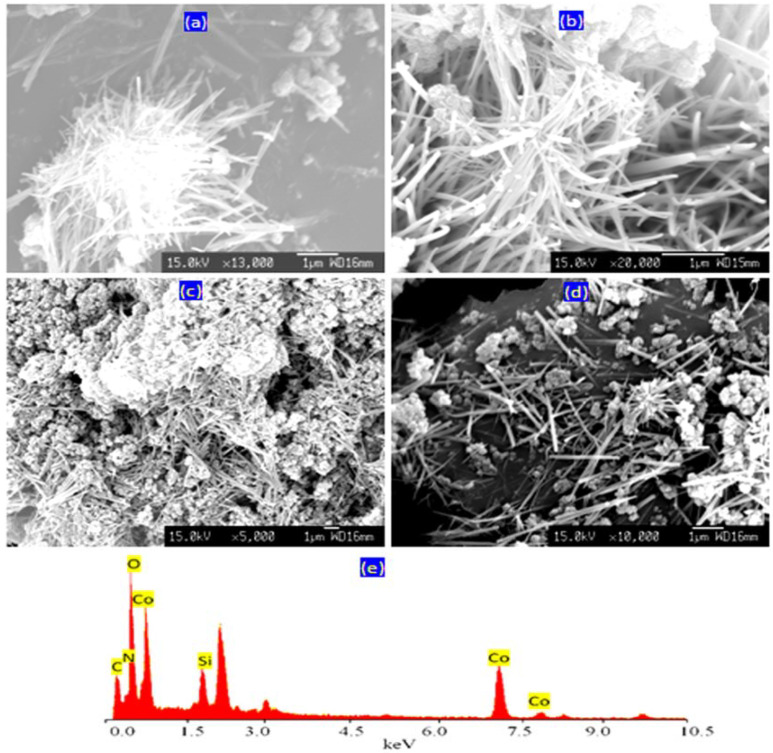
SEM images of samples (**a**) SiCo-1-16; (**b**) SiCo-1-26; (**c**,**d**) SiCo-2-36; and (**e**) EDX analysis of SiCo-1-26.

Scanning electron microscopy is an important technique for determining the morphology of Si/Co LDHs. In order to obtain clear images, the samples were coated by a thin film of platinum before measurements. However, whilst the SEM images of the natural samples of layered double hydroxides showed platy morphology, the current samples of Si/Co LDH exhibited an alternative morphology. Figure 1a shows the SEM images of sample Si/Co-1-16. It indicates that the sample Si/Co-1-16 has fibers in the nano scale. In addition, there are a few nanoparticles being observed in Figure 1a. These results were confirmed through measuring the SEM images of sample Si/Co-1-26 as shown in Figure 1b and Figure S1. Clear fibrous morphology was observed in a wide area of the sample Si/Co-1-26, as seen in Figure S1a. By magnifying, it showed clear nanofibers in the shape of aggregates and bundles, as seen in Figure S1b,c. In the other locations, the individual nanofibers were observed, indicating that the fibers of Si-Co-1-26 are in the nanoscale with a diameter of 50 nm, as shown in Figure S1c,d.

By increasing the molar ratio of Si/Co from 0.25 to 0.33, SEM images of Si/Co-2-36 showed nanoparticles in addition to a fibrous structure, as shown in Figure 1c,d. Additionally, Figure 1c revealed the aggregates of both nanofibers and the nanoparticles, together indicating the presence of two phases. The appearance of nanoparticles after increasing the content of silicon indicated that the extra silicon separated from the LDH structure to build new phase.

By comparing with the familiar plate-like morphology of LDHs, the nano-fibrous morphology of Si/Co LDH is unusual because the structure of LDHs consists of nanolayers with interlayered anions acting as pillars. In the usual LDHs, it is logical because the nanolayers of the majority of LDHs are composed of di- and tri-valent cations. Therefore, one positive charge (+1) was produced and neutralized with one negative charge (one pillar or one anion). However, in our study, the tetravalent silicon was used inside the nanolayers with cobalt. Therefore, a positive charge (+2) was produced and neutralized by two anions. Therefore, the nanofibers were formed through a mechanism which will be explained later in the subsequent sections.

Energy-dispersive X-ray spectrometry (EDX) analysis has provided clear information of the different elements in the outermost layers of the nanofibers of LDHs. Cobalt and silicon in addition to oxygen were detected in the wide area of the SEM image of SiCo-1-26 as shown in Figure 1e. Additionally, the signals of both nitrogen and carbon are clearly observed in Figure 1e, indicating the presence of cyanate and nitrate anions.

X-ray photoelectron spectroscopy (XPS), which is also known as electron spectroscopy for chemical analysis (ESCA), was used for measuring the oxidation state of cobalt in sample SiCo-1-26. According to the electron binding energy of Co (2p_3/2_) for sample SiCo-1-26, it was 785 eV, as shown in Figure S2. By comparing with the binding energy of cobalt in CoO (783–781 eV) [37,38] and in Co_2_O_3_ (781–779 eV) [37,38], the presence of cobalt as divalent cations was confirmed because the value 785 eV was near to the divalent cation (783 eV) and far from the trivalent cation (781 eV).

### 3.2. Fourier Transform Infrared Spectroscopy

**Figure 2:**
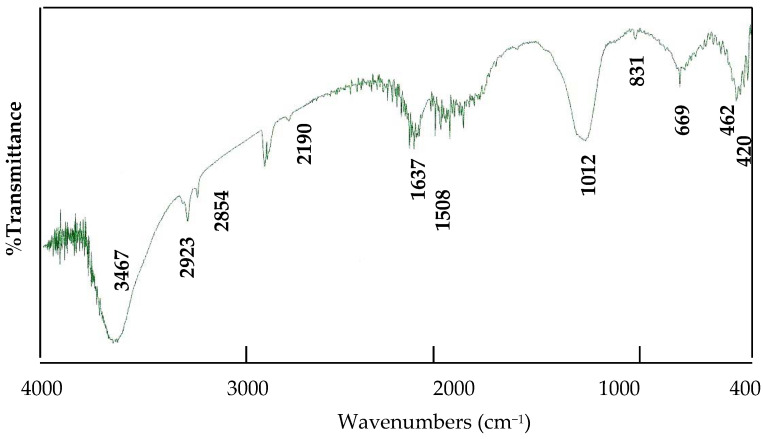
Infrared spectrum of the sample SiCo-1-26.

Fourier transform infrared spectroscopy was utilized to detect the interlayered anions of Si/Co LDH and determine its functional groups. Figure 2 showed the FT-IR spectrum of the nanofibers of Si/Co LDHs. 

Fourier transform infrared spectroscopy was utilized to detect the interlayered anions of Si/Co LDH and determine its functional groups. Figure 2 showed the FT-IR spectra of the nanofibers of Si/Co LDHs. The clear absorption bands, which were observed at 3467 cm^−1^ and 1637 cm^−1^, confirmed the presence of both the hydroxyl groups and the interlayered water. Additionally, Figure 2 showed small bands at 2923 cm^−1^ and 2854 cm^−1^, indicating the formation of hydrogen bonds between the interlayered water and anions. These characteristic bands confirmed the formation of the LDH structure. The absorption band of the interlayered cyanate anions was observed at 2190 cm^−1^. Additionally, the peak at 1012 cm^−1^ which was due to stretching mode of C–N was confirmed the presence of cyanate anions. In addition, the presence of nitrate anions was confirmed by two bands at 1508 cm^−1^ and 1384 cm^−1^. Additionally, the vibrational mode of nitrate (υ_4_) was observed at 669 cm^−1^. Characteristic peaks for O–Si–O appeared as a broad peak at 1012 cm^−1^ while the band observed at 462 cm^−1^ is due to Si–O–Co [39], as shown in Figure 2.

### 3.3. Powder X-ray Diffraction

**Figure 3:**
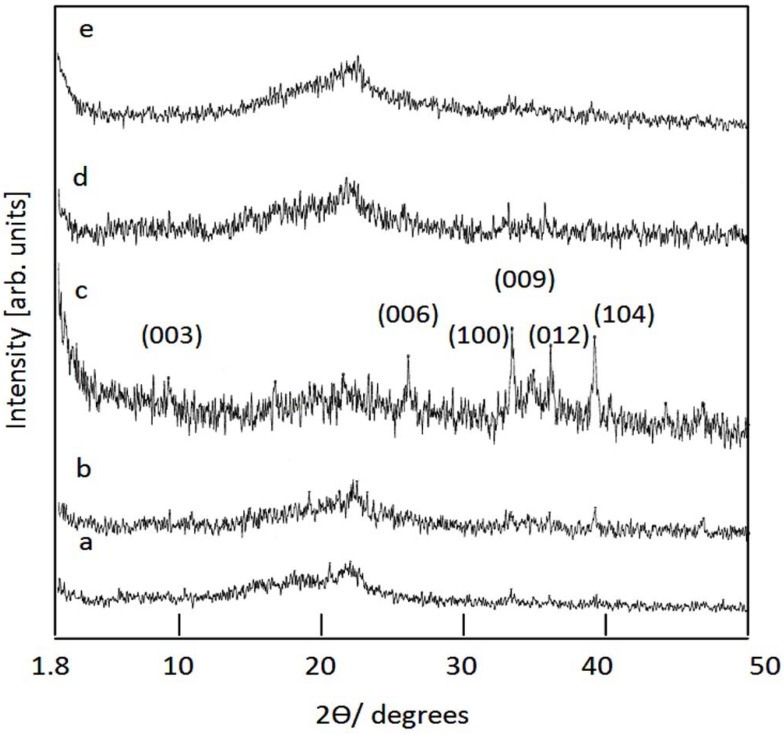
X-ray diffraction of Si/Co LDH: (a) SiCo-1-16; (b) SiCo-1-20; (c) SiCo-1-26; (d) SiCo-1-36; and (e) SiCo-2-36.

Figure 3 displays the X-ray diffraction patterns of the prepared products after the different reaction times 16–36 h. Figure 3(a) showed that the sample SiCo-1-16, which precipitated after reaction time 16 h, has a non-crystalline structure. 

In case the reaction time increased to 20 h, weak peaks started to grow as shown in Figure 3(b). During a reaction time lasting between 26 h and 36 h, the weak peaks became more observable in the diagram of sample SiCo-1-26, as shown in Figure 3(c). The main peaks of the layered double structure were observed at 0.79 nm, 0.36 nm and 0.26 nm, indicating the reflections of planes [003], [006], and [009], respectively. According to the spacing for plane [003], the parameter (c) was calculated to be 2.37 nm. By comparing with the (c) value of the synthetic and natural LDHs, a little shift was observed for the prepared Si/Co LDHs. Additionally, other peaks were observed at a spacing of 0.27 nm, 0.26 nm, 0.25 nm and 0.23 nm. According to Gastuche et al. and Saber et al. [40,41], these peaks agree with the diffuse non-basal reflections of the planes (100), (101), (012) and (104) of an LDH structure and confirm that the Si/Co LDHs has disordered structure. These data agree with the results which are previously published for Zn-Si LDH [41] and concluded that the presence of silicon inside the nanolayers of LDHs caused the distortion of the nanolayered structures of LDHs. 

By increasing the molar ratio of Si/Co, an amorphous structure was observed for the sample Si/Co-2-36, as shown in Figure 3(e). This means that the presence of a high silicon content caused strong distortion for the layered structure because of creating a new phase, which is consistent with the SEM results. 

**Figure 4:**
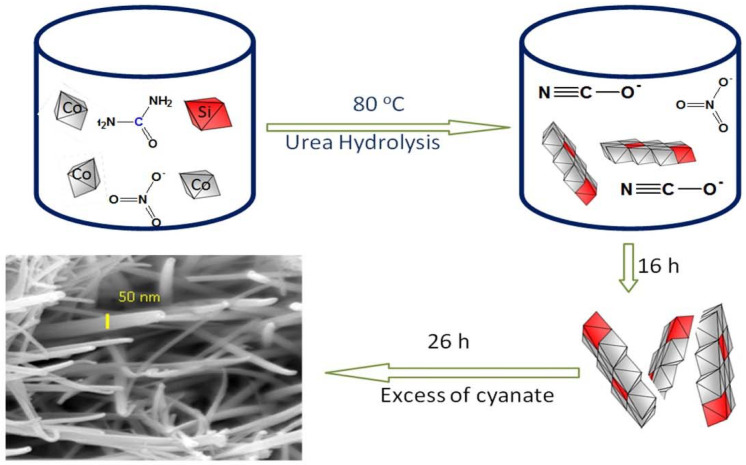
Schematic representation of the synthesis of 1D nanofibers.

According to the SEM images and XRD results, in addition to the XPS and EDX spectra, the effect of reaction and aging time plays an important role for building the nanofibers of Si/Co LDHs. Figure 4 shows the schematic representation of the synthesis of 1D nanofibers.

### 3.4. Thermal Analyses

**Figure 5:**
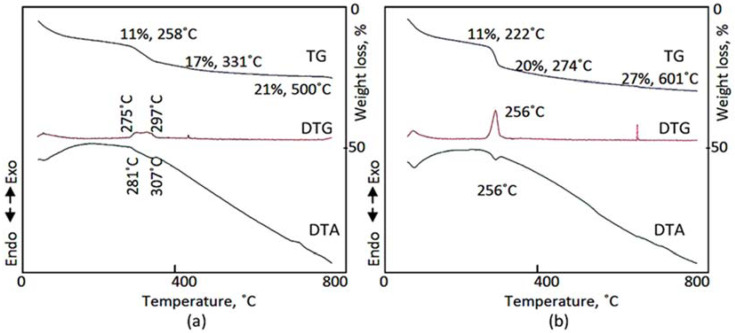
Thermal analyses of: (**a**) SiCo-1-16; and (**b**) SiCo-1-36.

The thermal behavior of samples SiCo-1-16 and SiCo-1-36 was determined by measuring the thermal gravimetric (TGA), differential thermal gravimetric (DTG) and differential thermal analyses (DTA). Figure 5a showed that the total weight loss of SiCo-1-16 was 21% and accomplished after heating at 500 °C, while the total weight loss of SiCo-1-36 was 27% and observed at 601 °C, as shown in Figure 5b. This means that the Si/Co LDHs needed more reaction time than 16 h to be completely formed. By studying the details of thermal analyses, the TG diagrams revealed that the intercalated water and surface water were lost through two stages in a similar way for both samples SiCo-1-16 and SiCo-1-36. At 100 °C, the surface water of both samples was easily lost and was 7–8 wt%, which is consistent with the clear peak in the DTG curve. 

The DTA curve confirmed that by observing the endothermic peak at 80 °C, the water molecules which strongly bonded with the interlayered anions were lost at higher temperatures of 222–258 °C for both samples and were 3–4 wt%. The decomposition of the interlayered anions of sample SiCo-1-16 happened in two steps. The first step was 6 wt% and occurred at 331 °C. The second step was 4 wt% and took place at 500 °C. The DTG curve revealed two peaks at 275 °C and 297 °C, confirming the presence of two interlayered anions. Additionally, the dual interlayered anions were confirmed by DTA curve. However, in the case of sample SiCo-1-36, the first anion 9 wt% was sharply lost at 274 °C, exhibiting an endothermic peak in the DTA curve and a sharp peak in the DTG curve at 256 °C. Meanwhile, the second anion 7 wt% was gradually lost at up to 601 °C with the dihydroxylation process of the nanolayers. These results concluded that the nanofibers of Si/Co LDH formed after 26 h of reaction time. During this time, the dual anions of cyanate and nitrate intercalated with a large amount of water inside the interlayered space.

### 3.5. Formation Mechanism of One-Dimensional Nanofibers

The usual geometry and morphology of Co-Al LDHs are two-dimensional layered structures with a hexagonal shape [42]. In the current structure, a one-dimensional structure was observed for Si/Co LDHs. According to the difference between the usual LDHs and the current LDHs, the mechanism of conversion from a 2D material to a 1D structure can be applied for explaining this behavior. The current Si/Co LDH creates cationic nanolayers with positive charges (+2) on silicon because of the combination between the divalent cobalt and the tetravalent silicon. In addition, the FI-IR results and thermal analyses confirmed the presence of cyanate anions (CNO^−^) and nitrate anions which can be used as pillars for building the LDH structure and neutralizing the positive charges (+2) of the nanolayers of Si/Co. These cyanate anions were produced through urea hydrolysis while the nitrate anions were released from the precursor of cobalt. Urea is a very weak Bronsted base (pK_b_ = 13.8). By controlling the temperature, urea is slowly hydrolyzed and decomposed to form ammonium cyanate, converting the medium of the reaction from an acidic nature (pH = 3) to alkaline nature (pH = 8).

**Figure 6:**
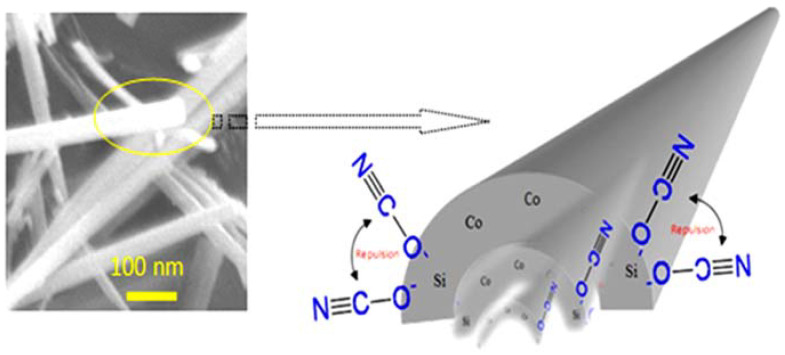
Schematic representation of 1D nanofiber.

This process needs a long time to produce enough amounts of cyanate anions in addition to achieving an alkaline medium for precipitating LDHs. Therefore, SEM images showed that the better quality of nanofibers was obtained after 26 h of the reaction. The positive charges (+2), which produced from the combination between cobalt and silicon, attracted the cyanate anions and/or nitrate anions which were identified by FTIR and thermal analyses. The comparison between the structure and the size of both nitrate and cyanate anions indicated that the intercalation of cyanate anions to build LDHs is more favorable because of its straight structure. By competition with the nitrate anions, the pull of two cyanate anions (−1) toward the silicon cation (+2) is occurred through only one side as shown in Figure 6. The steric hindrance between these two anions created strong repulsion forces inside the interlayered region of LDH. These forces pushed and pressed on the nanolayers, especially on their edges leading to curling and curving for the nanolayers producing nanofibers as shown in Figure 6.

### 3.6. Electrochemical Studies

**Figure 7:**
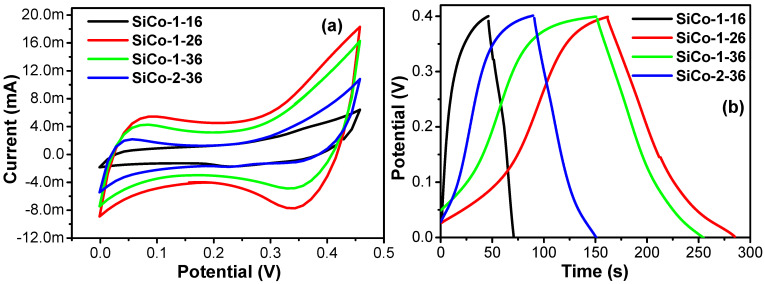
Comparative (**a**) CV curves of the SiCo-1-16, SiCo-1-26, SiCo-1-36, and SiCo-2-36 LDH electrodes at fix scan rate of 5 mV/s; and (**b**) CD curves of SiCo-1-16, SiCo-1-26, SiCo-1-36, and SiCo-2-36 LDH electrodes at fixed current density of 2 A g^−1^.

Electrochemical supercapacitor properties of the nano size one-dimensional Si/Co LDH samples explored in three-electrode system. The system based on one-dimensional Si/Co LDHs as a working electrode, platinum sheet as a counter electrode, Ag/AgCl as a compared electrode, and 2 moles of KOH electrolyte. A collection of the effective electrochemical method such as cyclic voltammetry (CV) and Galvano static charge–discharge (GCD) applied to investigate the electrochemical properties of the SiCo-1-16, SiCo-1-26, SiCo-1-36 and Si/Co-2-36 LDH electrode materials. The voltage range of the optimal capacitance of the Si/Co LDHs was measured by the CV curves under the scan rate of 5 mV/s and the optimal range was from 0.0 V to 0.45 V (Figure 7a). 

Figure 7a represents the comparative CV graphs of the SiCo-1-16, SiCo-1-26, SiCo-1-36 and Si/Co-2-36 LDH electrodes at a fixed scan rate, which reveals the impact of the synthesis time duration and mole ratio of Si/Co on their electrochemical properties. All four samples of Si/Co LDH with a different synthesis time duration and different mole ratio of Si/Co show regular CV curves and all four samples have a couple of redox peaks between approximately 0.05 and 0.35 V at a fixed scan rate of 5 mV/s, which indicates the faradic nature [43,44]. It is well known that the charges are stored with respect to the area under the CV curves. The redox peak assigned the reaction of Co^2+^/Co^3+^ in the Si/Co LDH in alkaline electrolytic solution [44,45,46]. From CV curves, it can be seen that the SiCo-1-26 sample’s large integrated area in the CV curve as compared to other samples of Si/Co LDH indicates a higher specific capacitance provided by the SiCo-1-26 sample electrode. This enhanced performance of the SiCo-1-26 LDH electrode is due to the nano fibrous morphology of the material, which secondly might be due to the synergetic effect between silicon as EDLC and the pseudocapacitive nature of cobalt. Changing the synthesis time duration also affects the morphology of the electrode material, which affects the specific capacitance of the electrode material. In the case of the SiCo-1-36 electrode, which was due to a long synthesis time, the nano fibers became thick, which is responsible for the decrease in the capacitance of the electrode material (SiCo-1-36). In the case of SiCo-2-36 from the SEM images, it can be clearly seen that the nanoparticles adhere to the nanofibers and both nanofibers and nanoparticles aggregate together, hindering the capacitive performance of the SiCo-2-36 electrode. 

The comparative GCD data are also proposed in Figure 7b at the current density of 2 A/g^−1^ and in the potential range of the 0.0 to 0.4 V. All the electrodes demonstrate rapid response and excellent electrochemical reversibility, confirming the faradic behavior of electrodes. Moreover, the specific capacitance of these samples calculated from the length of charge/discharge curve and from Equation (S1) mention inside the electronic supplementary information of the Si/Co LDH electrodes confirm this result precisely. The SiCo-1-16, SiCo-1-26, SiCo-1-36 and Si/Co-2-36 LDH electrodes depicted the highest calculated specific capacitance of 140 F g^−1^, 621.5 F g^−1^, 515 F g^−1^ and 326.5 F g^−1^ at the current density of 2 A g^−1^ [47,48], respectively. The SiCo-1-26 LDH electrode has the largest specific capacitance (621.5 F g^−1^) as compared to the SiCo-1-16 (140 F g^−1^), SiCo-1-36 (515 F g^−1^) and Si/Co-2-36 (326.5 F g^−1^) LDH electrodes; moreover, the calculated CD result coordinates well with the CV result.

**Figure 8:**
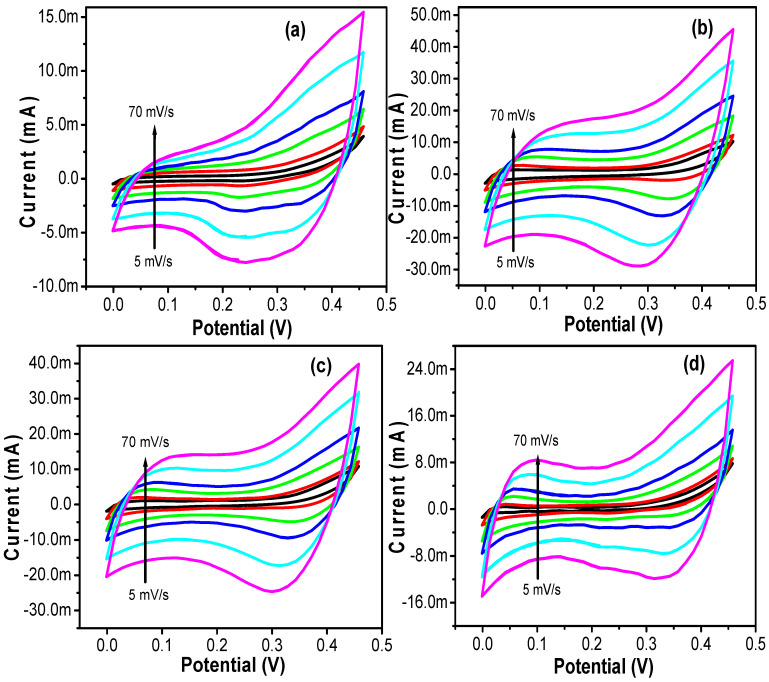
CV curves of the (**a**) SiCo-1-16; (**b**) SiCo-1-26; (**c**) SiCo-1-36; and (**d**) SiCo-2-36 LDH electrodes at different scan rates.

Figure 8 depicted the CV curves of all the electrode samples SiCo-1-16, SiCo-1-26, SiCo-1-36 and Si/Co-2-36 LDH, respectively, at different scan rates between 5 mV s^−1^ and 70 mV s^−1^, and within the potential range of 0.0–0.45 V verses Ag/AgCl standard electrode. CV curves of all the samples show the redox peak or redox activity on the electrode surface, which clearly indicates the pseudocapacitor behavior of the electrode material. Moreover, from the CV curves, the oxidation–reduction peaks can be observed, which move towards to the higher and lower potential due to the reinforced electric polarization and feasible kinetic irreversibility of the electrolytic ion on the electrode surface. The electrode material which displayed the redox peak in the CV analysis should not be presumed to be a pseudocapacitor, as the electrode keep altering over the whole potential window. Consequently, the electrochemical supercapacitive performance of all the Si/Co LDH electrodes were measured in terms of the specific capacity instead of specific capacitance [27].

**Figure 9:**
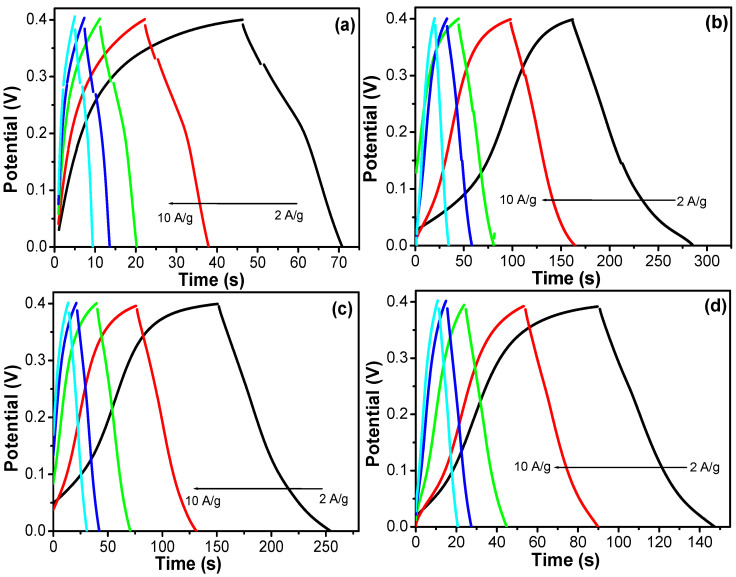
GCD curves of the (**a**) SiCo-1-16; (**b**) SiCo-1-26; (**c**) SiCo-1-36; and (**d**) SiCo-2-36 LDH electrodes at different current densities.

For better applicability, the electrochemical behavior of the prepared electrodes was directly evaluated by the galvanostatic charge–discharge (GCD) method at different current densities. Figure 9 shows the CD curves of the SiCo-1-16, SiCo-1-26, SiCo-1-36 and Si-Co-2-36 LDHs electrodes at the current densities ranging from 2 A g^−1^ to 10 A g^−1^. The calculated specific capacitance of the electrode material with respect to the altering synthesis time duration are as follows: in the case of SiCo-1-16, the LDH electrodes at 2, 3, 5, 7 and 10 A g^−1^ are 140, 120, 125, 119, 112.5 F g^−1^, the SiCo-1-36 LDH electrode at 515, 427.5, 400, 350, 325 F g^−1^ and in the case of the SiCo-1-26 LDH electrode, the estimated specific capacitances are approximately 621.5, 510, 475, 437.5, 375 F g^−1^, respectively. As compared to the SiCo-1-16 and SiCo-1-36 LDH electrodes, the SiCo-1-26 LDH electrode delivered a long time charge/discharge performance due to its unique (nano size particle mixed fibrous) morphology, which provided the larger surface area and more active sites for the electrolyte intercalation/deintercalation during the CD process and maximized the utilization of the SiCo-1-26 LDH electrode. 

**Figure 10:**
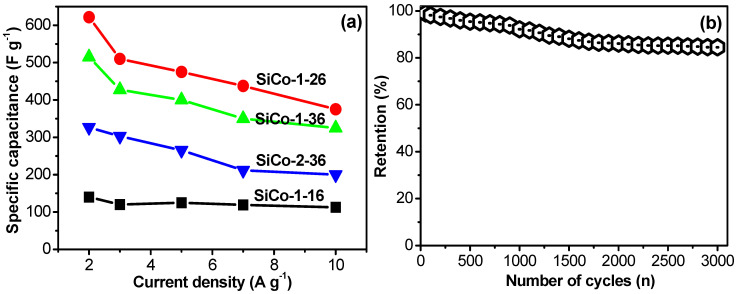
(**a**) Calculated specific capacitance of the SiCo-1-16, SiCo-1-26, SiCo-1-36 and Si/Co-2-36 LDH electrodes at different current densities; and (**b**) cyclic stability of SiCo-1-26 LDH electrodes over 3000 cycles.

To understand the effect of the ratio of the Si and Co material on the morphology and electrochemical performance of Si/Co LDH electrodes, the CV and GCD data profile of the SiCo-2-36 LDH electrode are proposed in Figure 6. From the CV curve (Figure 7a), it is clearly shown that the sample shows a small integrated area which displays that the capacitance of SiCo-2-36 LDH electrode decreased. The CD curve (Figure 7b) also depicted the lower performance of the SiCo-2-36 LDH electrode due to the aggregation of the nanoparticles and nanofibers. The specific capacitance of the SiCo-1-16, LDH electrode at 2, 3 5, 7 and 10 A g^−1^ are 326.5, 303, 265, 211.75, 200 F g^−1^, respectively. Figure 10a represents the calculated specific capacitance of the prepared electrodes (SiCo-1-16, SiCo-1-26, SiCo-1-36 and Si-Co-2-36 LDHs electrodes) at different current densities. From Figure 10a, it clearly shows that with the increasing current density, the specific capacitance of the synthesized electrodes decreased due to the decreased penetration of the electrolyte at higher current.

The long-term stability performance of the electrode material is one of the most important concerns in energy storage applications. Generally, metal oxides suffer from the poor cyclic stability because of degradation [49,50]. The cyclic stability test and the coulombic efficiency (Figure S3) of the optimized SiCo-1-26 LDH electrode were analyzed by the GCD analysis at 5 A g^−1^ for continuous 3000 charge/discharge cycles. From Figure 10b, it is clearly shown that in the 400 initial cycles, the capacitance of the electrode material rapidly decreases due to the active site saturation of the surface of electrodes during the charge–discharge mechanism stability of the electrode. As shown in Figure 10b, the nano size particle mixed fibrous retained 84.5% of the specific capacitance after 3000 cycles. The better performance of the SiCo-1-26 LDH electrode material was due to the nano size particle mixed fibrous morphology, which provided more active sites during the electrochemical test and helped stabilize the overall structure of the nanocomposite during the continuous charging–discharge process up to 3000 cycles.

## 4. Conclusions

In the current study, a dual objective was attained by preparing the Si/Co nanofibers and obtaining a new candidate for supercapacitor electrodes. The SEM images showed that the prepared Si/Co LDHs, which were prepared after a reaction time of 26 h, had nanofibers which were 50 nm in diameter. Additionally, the X-ray diffraction, FTIR and thermal analyses showed that these LDHs have two interlayered anions; cyanate and nitrate. The steric hindrance between the two bulky anions inside the interlayered region of LDHs led to strong repulsion forces between them, causing curling for the nanolayers of LDH-producing nanofibers. 

The electrochemical characterization indicated that the change of the plate-like morphology, which is the familiar morphology of LDHs, to that of nanofibers, had the positive effect of improving the performance of the specific capacitance of Si/Co LDHs. The specific capacitance increased to 621.5 F g^−1^ in the case of the nanofiber of Si/Co LDHs. In addition, the excellent cyclic stability arrived to 84.5%. Furthermore, the nanofibers helped stabilize the overall structure of the nanocomposite during the continuous charging–discharge process up to 3000 cycles. Finally, the high capacitance of the ternary system of the Si/Co/cyanate nanofibers was suggested to be used as active electrode materials for high-performance supercapacitors applications. 

## Data Availability

Data are available in a publicly accessible repository.

## Supplementary Materials

The following supporting information can be downloaded at: https://www.mdpi.com/article/10.3390/nano12091404/s1, Figure S1: SEM images of the sample SiCo-1-26 at different magnification (a) 10 µm, (b) 100 nm, and (c,d) 50 nm; Figure S2: XPS analysis for SiCo-1-26; Figure S3: Coulombic efficiency profile of the optimized electrode examined at current density of 7 A g^−1^.
