# Supplementary material for: One-Dimensional Nanoscale Si/Co Based on Layered Double Hydroxides towards Electrochemical Supercapacitor Electrodes
Source: Nanomaterials (Basel). 2022 Apr 20;12(9):1404. doi: 10.3390/nano12091404 (PMC9101559; doi:10.3390/nano12091404)
Supplement: Supplementary file 1 [file nanomaterials-12-01404-s001.zip › nanomaterials-1662190-supplementary.pdf]

nanomaterials

MDPI

Electronic Supplementary Information (ESI)

One-Dimensional Nanoscale Si/Co Based on Layered Double Hydroxides towards Electrochemical Supercapacitor Electrodes

Osama Saber [1,2,*], Sajid Ali Ansari [1,*], Aya Osama [1] and Mostafa Osama [1]

1 Department of Physics, College of Science, King Faisal University, P.O. Box 400, Al-Ahsa 31982, Saudi Arabia; 217044956@student.kfu.edu.sa (A.O.); 214110595@student.kfu.edu.sa (M.O.)
2 Egyptian Petroleum Research Institute, Nasr City, P.O. Box 11727, Cairo 11765, Egypt
* Correspondence: osmohamed@kfu.edu.sa (O.S.); sansari@kfu.edu.sa (S.A.A.); Tel.: +966-13-589-9440 (O.S.)

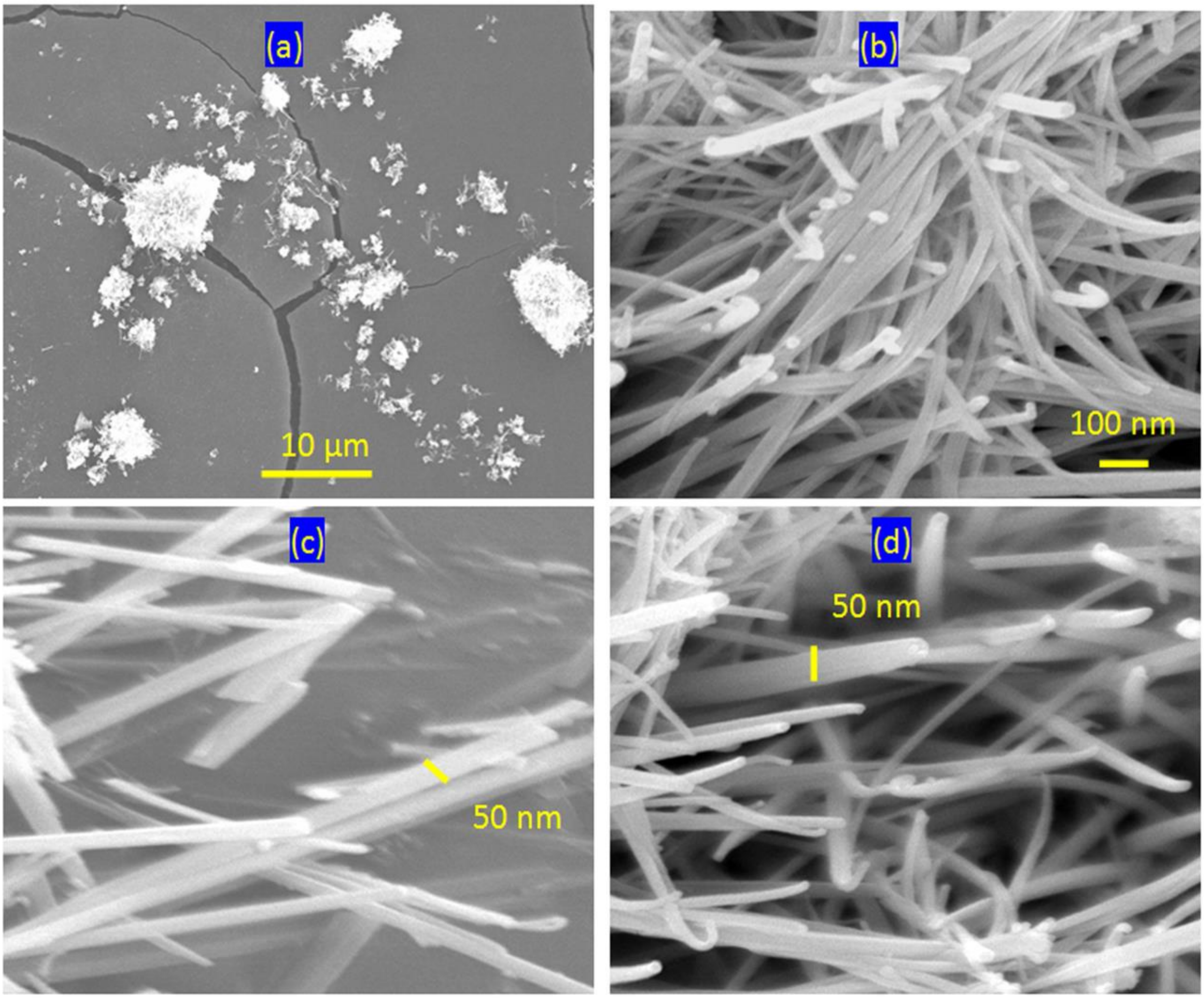

Figure S1. SEM images of the sample SiCo-1-26 at different magnification (a) 10 μm, (b) 100 nm, and (c,d) 50 nm.

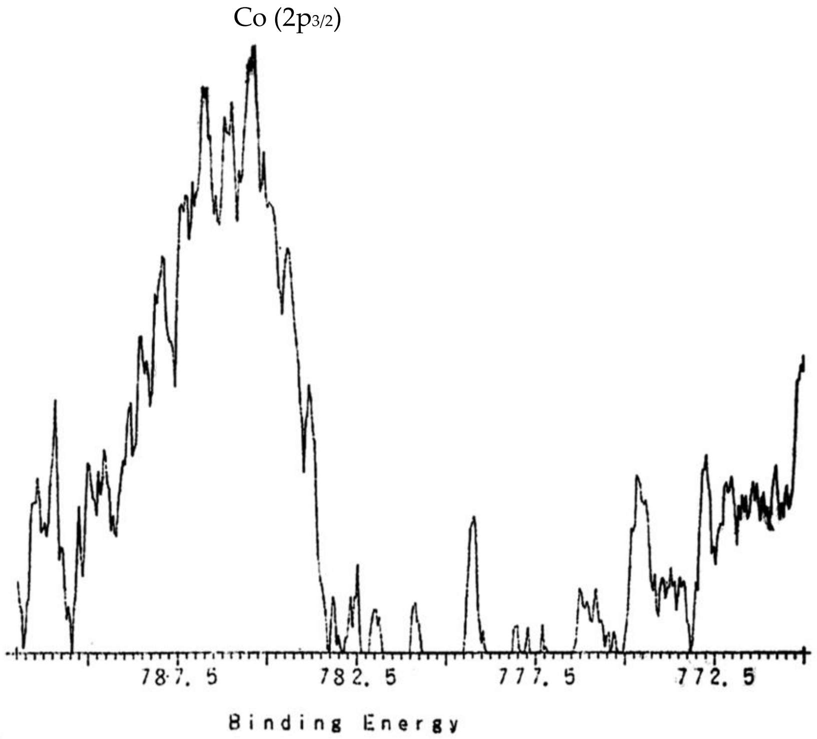

Figure S2. XPS analysis for SiCo-1-26.

Specific capacitance

$C = Idt/mdV$ (S1)

where C represents specific capacitance (F g^{-1}), t represents the discharging time, m is the mass of the active materials coated/grown over the current collector, dV represents the applied potential window, and I represents the applied current and is the applied potential window.

Coulombic efficiency

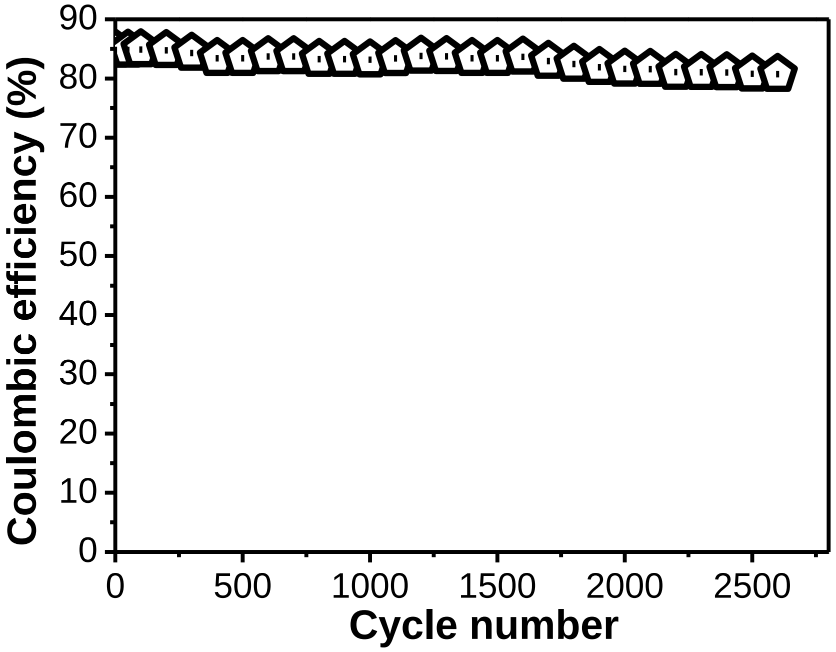

Figure S3. Coulombic efficiency profile of the optimized electrode examined at current density of 7 A g^{-1}.
